# *In Vivo* and *In Vitro* Antidiabetic Activity of *Terminalia paniculata* Bark: An Evaluation of Possible Phytoconstituents and Mechanisms for Blood Glucose Control in Diabetes

**DOI:** 10.1155/2013/484675

**Published:** 2013-07-14

**Authors:** Subramaniam Ramachandran, Aiyalu Rajasekaran, Natarajan Adhirajan

**Affiliations:** KMCH College of Pharmacy, Kovai Estate, Kalapatti Road, Coimbatore-641048, Tamil Nadu, India

## Abstract

The present study was aimed to investigate *in vivo*, *in vitro* antidiabetic activity of aqueous extract of *Terminalia paniculata* bark (AETPB) and characterize its possible phytoconstituents responsible for the actions. Type 2 diabetes was induced in rats by streptozotocin-nicotinamide (65 mg/kg–110 mg/kg; *i.p.*) administration. Oral treatment of AETPB using rat oral needle at 100 and 200 mg/kg doses significantly (*P* < 0.001) decreased blood glucose and glycosylated haemoglobin levels in diabetic rats than diabetic control rats. AETPB-treated diabetic rats body weight, total protein, insulin, and haemoglobin levels were increased significantly (*P* < 0.001) than diabetic control rats. A significant (*P* < 0.001) reduction of total cholesterol and triglycerides and increase in high-density lipoprotein levels were observed in type 2 diabetic rats after AETPB administration. Presence of biomarkers gallic acid, ellagic acid, catechin, and epicatechin in AETPB was confirmed in HPLC analysis. AETPB and gallic acid showed significant (*P* < 0.001) enhancement of glucose uptake action in presence of insulin in muscle cells than vehicle control. Also AETPB inhibited pancreatic **α**-amylase and **α**-glucosidase enzymes. In conclusion, the above actions might be responsible for the antidiabetic activity of AETPB due to presence of gallic acid and other biomarkers.

## 1. Introduction

Diabetes is characterized by hyperglycemia, altered lipids, carbohydrates, and proteins metabolism which affect the patient quality of life in terms of social, psychological well-being as well as physical ill health [1, 2]. Two forms of diabetes (Types 1 and 2) differ in their pathogenesis, but both have hyperglycemia as a common hall mark. In type 2 diabetes, hyperglycemia caused due to impairment in insulin secretion combined with or without impairment of insulin action [3]. The World Health Organization reported that worldwide global population is in the midst of a diabetes epidemic. The people in Southeast Asia and Western Pacific are being under greater risk, and the majority of patients have type 2 diabetes. Insulin resistance typically precedes the onset of type 2 diabetes and is commonly accompanied by other cardiovascular risk factors such as dyslipidemia, hypertension, and prothrombotic factors [4].

Diabetes-related cardiovascular complications occur due to altered lipoprotein metabolism-mediated atherosclerosis, and diabetics are 2 to 4 times more likely to suffer from stroke [5]. Although different classes of drugs are available to control type 2 diabetes, still it is a challenging task to bring a better molecule which is devoid of undesirable adverse effects than existing drugs. In Indian traditional medicine systems, the number of medicinal plants has been used since ancient time to effectively treat diabetes [6]. Multiple mechanisms, due to many phytoconstituents, were documented for the antidiabetic activity of medicinal plants. Therefore, documenting the efficacy of antidiabetic medicinal plants has been increased, and their characterizations of chemical constituents are focused in drug discovery programmes to bring a better lead molecule to treat diabetes [7].

*Terminalia paniculata *Roth. (Combretaceae) is a large deciduous tree distributed in the semievergreen and moist deciduous forests of India. In Ayurvedic system, bark was used to treat cough, strangury, diabetes, bronchitis, leprosy and skin diseases [8]. The hepatoprotective and anti-inflammatory activity of its bark was reported [9, 10]. Previously, we have reported antidiabetic and antioxidant activity of *Terminalia paniculata* bark in type 1 diabetes [11]. But, till date, there is no scientific report available to support the antidiabetic activity of *Terminalia paniculata* bark in type 2 diabetes and also its mechanisms responsible for the antidiabetic activity. Therefore, the present study was aimed to investigate *in vivo* antidiabetic activity of aqueous extract of *Terminalia paniculata* bark (AETPB) in type 2 diabetic rats. The phytoconstituents (biomarkers) present in AETPB were characterized by high-performance liquid chromatography (HPLC) analysis. The possible mechanisms responsible for the antidiabetic activity of AETPB were studied by *in vitro* methods such as glucose uptake activity in L6 rat skeletal muscle cells and inhibition of carbohydrate-metabolizing enzymes pancreatic *α*-amylase and *α*-glucosidase activity. 

## 2. Materials and Methods

### 2.1. Extraction of Plant Material

*Terminalia paniculata *was identified in Anaimalai hills, Coimbatore, Tamil Nadu, India and bark specimen was collected and authenticated by the Botanical Survey of India (BSI), Coimbatore (BSI/SRC/5/23/09-10/Tech.-813). Bulk collection of bark was carried out, and it was cleaned, shade-dried, and powdered. A 100 g of powdered bark was soaked in distilled water for 12 h and boiled for 30 min. The above aqueous solution was filtered, concentrated, and stored in an air-tight glass container. The yield of the extract was 11% w/w, and it was kept at 2–8°C until the completion of studies.

### 2.2. Experimental Animals

Male Wistar rats (150–200 g) were used to study the antidiabetic activity. Animals were housed in standard laboratory conditions (temperature 22 ± 2°C and humidity 45 ± 5°C with 12 h day: 12 h night cycle). All animals received standard laboratory diet and water *ad libitum*. Antidiabetic activity assessment was performed after the approval of Institutional animal Ethical Committee in accordance with institutional ethical guidelines for the care of laboratory animals of KMCH College of Pharmacy, Coimbatore, India (KMCRET/Ph.D/5/09). 

### 2.3. Chemicals

Streptozotocin and nicotinamide were procured from Himedia Laboratories, Mumbai, India. The estimation of biochemical parameters was carried out using kits (Primal Healthcare Limited, Lab Diagnostic Division, Mumbai, India). The pancreatic *α*-amylase was purchased from Sigma-Aldrich, USA. Dulbecco's-Modified Eagle's Medium (DMEM), *α*-glucosidase, Roswell Park Memorial Institute 1640 (RPMI1640), fetal bovine serum (FBS), trypan blue, potato starch, and maltose were procured from Himedia Laboratories, Mumbai, India. All other chemicals used in this study were analytical grade and purchased from Himedia Laboratories, Mumbai, India. The acetonitrile (HPLC grade), potassium dihydrogen orthophosphate, and orthophosphoric acid were purchased from RFLC Limited (Rankem), Mumbai, India. The standard gallic acid (GA), ellagic acid, catechin, and epicatechin were received from Natural Remedies Ltd, Bangalore, India, as gift samples.

### 2.4. *In Vivo* Study

#### 2.4.1. Experimental Type 2 Diabetes Induction

Type 2 diabetes was induced by injection of freshly prepared streptozotocin (STZ—65 mg/kg; *i.p.*) in cold citrate buffer (0.1 M, pH 4.5), 15 minutes after the administration of nicotinamide (NIC—110 mg/kg; *i.p.*) in overnight-fasted rats [2]. Diabetes induction was assured after 72 h by measurement of blood glucose levels by glucose meter (Glucocard 01-mini, Arkray Factory Inc., Japan). To stabilize the blood glucose level, diabetic rats were kept under standard laboratory condition up to 14 days. Blood glucose was again determined on day 14, and diabetic rats showing blood glucose >200 mg/dL were selected to assess the antidiabetic activity of AETPB. 

#### 2.4.2. Study Design for Assessment of AETPB *In Vivo* Antidiabetic Activity

To assess the antidiabetic activity of AETPB the following groups were made, and each group consists of six rats. Group 1: normal control rats received 0.2% carboxy methyl cellulose (CMC; 5 mL/kg). Group 2: diabetic rats received 0.2% CMC (5 mL/kg). Group 3: diabetic rats received AETPB at 100 mg/kg dose. Group 4: diabetic rats received AETPB at 200 mg/kg dose. Group 5: diabetic rats received glibenclamide at 5 mg/kg dose [12]. AETPB dose was selected based on our previous study findings [11], and vehicle, AETPB, and glibenclamide were administered by rat oral needle to their respective group of rats up to 28 days. All samples (AETPB in water and glibenclamide in 0.2% CMC) were prepared freshly before the oral administration on each day. The fasting blood glucose level and body weight were estimated at 14 and 28 days after the treatments. On day 28, vehicle, AETPB and glibenclamide were administered to the overnight-fasted rats, and after 1 h treatments all animals were anaesthetized with ketamine (100 mg/kg, *i.p.*). Blood samples were obtained by puncture of retro-orbital plexus and stored with or without disodium ethylene diamine tetra acetate to evaluate the biochemical parameters. 

#### 2.4.3. Measurement of Biochemical Parameters

The blood glucose, Hb, and HbA1c were estimated using whole blood. The serum total cholesterol (TC), high-density lipoprotein (HDL), triglycerides (TG), and total protein (TP) were estimated using commercially available kits in a semiautoanalyzer (Photometer 5010_V5+_, Germany). The serum insulin was estimated by radioimmunoassay method.

### 2.5. Characterization of Biomarkers by HPLC Analysis

Chromatographic analysis was carried out in HPLC (LC2010AHT, Shimadzu, Japan; quaternary pump, dual channel UV detector, autosampler, and column oven) by reversed-phase C-18 end-capped column (Phenomenex-Luna C18 (2); ø 250 mm × 4.6 mm) packed with 5 *μ*m diameter particles. The standard gallic acid (100 *μ*g/mL), ellagic acid (120 *μ*g/mL), catechin, epicatechin (160 *μ*g/mL), and AETPB (2670 *μ*g/mL) were used in this study, and analysis was performed by gradient method. The mobile phase, phosphate buffer (1 mM, pH 2.8; solvent A) and acetonitrile (solvent B), was used with flow rate of 1.50 mL/min at 25°C up to 45 min, and AETPB (20 *μ*L) was injected into the column. 

The presence of phytoconstituents such as GA, catechin, and epicatechin was detected at 270 nm, and ellagic acid was detected at 370 nm by UV detector following their separation. The AETPB phytoconstituents were confirmed by comparison of its retention time with standard phytoconstituents retention time. After the analysis, weight, purity, peak area of the standard, and AETPB phytoconstituents were used to estimate the amount (%w/w) of constituents present in the AETPB. 

### 2.6. *In Vitro* Studies

#### 2.6.1. Cytotoxicity Study of AETPB and GA in L6 Rat Skeletal Muscle Cells

Cytotoxicity study was carried out using L6 rat muscle cells with final density of 1 × 10^5^ cells/mL. Cell suspension (100 *μ*L per well) was seeded into a 96-well plate at plating density of 10,000 cells per well and incubated at 37°C, 5% CO_2_, 95% air, and 100% relative humidity. After 24 h, cells were treated with five concentrations of AETPB (12.5, 25, 50, 100, and 200 *μ*g/mL) and five concentrations of GA (6.25, 12.5, 25, 50, and 100 *μ*M) in serum free medium. Aliquots of 100 *μ*L of the above concentrations of AETPB and GA were added to the appropriate wells, which already contained 100 *μ*L of medium, to make up to final sample concentrations. After the addition of AETPB and GA, plate was incubated for 48 h at 37°C, 5% CO_2_, 95% air and 100% relative humidity. The control well contains medium with AETPB and GA without muscle cell, and all concentrations of AETPB and GA were performed in triplicates. Cytotoxicity of AETPB and GA was assessed by MTT (3-(4,5-dimethylthiazolyl-2)-2,5-diphenyltetrazolium bromide) assay, and percentage cell viability was calculated.

#### 2.6.2. Glucose Uptake Assay in L6 Rat Skeletal Muscle Cell

L6 rat myogenic cells were cultured in DMEM containing 4.5 g/L D-glucose with 10% heat-inactivated FBS at 37°C, 5% CO_2_ atmosphere. The cells were seeded into 96-well plate with six wells left as blank wells and let growing to confluence; then cells were fully differentiated in DMEM with 2% FBS for 5 days. Before tests, the medium was replaced by RPMI1640 (2 g/L glucose) supplemented with 0.2% BSA. The medium was removed after 2 h, and the same medium containing AETPB (0.5, 5 and 10 *μ*g/mL), GA (0.05, 0.5 and 5 *μ*M), metformin (0.01 mM), and DMSO in absence or presence of insulin (1 *μ*mol/L) was added to all wells including the blank. The glucose in the medium was determined by the glucose-oxidase method after 48 h treatment [13]. The amount of glucose uptake by muscle cells was calculated by using the following formula:

(1) Glucose  uptake =[Glucose  concentration  of  blank  wells]  −[Glucose  concentration  of  cell plated  wells].

#### 2.6.3. Inhibition of *α*-Amylase Activity

Starch solution (0.5% w/v) was prepared by stirring potato starch (0.125 g) in 20 mM sodium phosphate buffer with 6.7 mM sodium chloride (pH 6.9; 25 mL) in a boiling water bath for 15 min. The *α*-amylase solution was prepared by mixing 1 U/mL of *α*-amylase in the same buffer. The colorimetric reagent was prepared by mixing equal volume of sodium potassium tartrate tetrahydrate solution and 96 mM 3, 5-dinitro salicylic acid (DNS) solution. Starch solution (1000 *μ*L) was mixed with increasing concentration of an enzyme inhibitor such as AETPB (1, 2, 4, 6, 8, and 10 *μ*g/mL) or acarbose (50–1000 *μ*g/mL), and to this 1000 *μ*L of *α*-amylase solution was added and incubated at 25°C for 3 min to react with the starch solution. A 1000 *μ*L of 96 mM DNS reagent was added to the above solution, and the contents were heated for 15 min on a boiling water bath. The final volume was made up with distilled water, and the absorbance was measured at 540 nm using spectrophotometer [14]. The percentage inhibition and 50% inhibitory concentration (IC_50_) value was calculated. 

#### 2.6.4. Inhibition of *α*-Glucosidase Activity

The *α*-glucosidase enzyme inhibition activity was determined by incubating 100 *μ*L of *α*-glucosidase enzyme (1 U/mL) solution with 100 *μ*L of phosphate buffer (pH 7.0) which contains 100 *μ*L of enzyme inhibitor such as AETPB (25–1600 *μ*g/mL) or acarbose (0.1–3.2 *μ*g/mL) at 37°C for 60 min in maltose solution. To stop the *α*-glucosidase action on maltose, the above reaction mixture was kept in boiling water for 2 min and cooled. To this, 2 mL of glucose reagent was added and its absorbance was measured at 540 nm to estimate the amount of liberated glucose by the action of *α*-glucosidase enzyme [14]. The percentage inhibition and 50% inhibitory concentration (IC_50_) value was calculated. 

### 2.7. Statistical Analysis, Determination of Percentage Inhibition and IC_50_ Value

In animal study, data were expressed as mean ± SEM. The statistical analysis was carried out using one-way analysis of variance (ANOVA), followed by Dunnett's test for multiple comparisons, and values of *P* < 0.05 were considered as statistically significant. 

The percentage inhibition of *α*-amylase and *α*-glucosidase was calculated using the following formula:

(2) Percentage  inhibition =Absorbance  of  control−Absorbance  of  testAbsorbance  of  control×100.

*In vitro* data were expressed as mean percentage inhibition ± SD (*n* = 3). IC_50_ value of percentage inhibition of enzymes was determined using nonlinear regression graph (log_10_ concentration versus percentage enzyme inhibition). In glucose uptake activity, statistical significance between groups was determined by one-way analysis of variance, followed by Dunnett's test for multiple comparisons, and *P* < 0.05 was considered as statistically significant. All statistical analysis and IC_50_ value determination were carried out in GraphPad Prism (Version 5.0) software.

## 3. Results 

### 3.1. *In Vivo* Study

#### 3.1.1. Blood Glucose and Body Weight Changes in Type 2 Diabetic Rats

**Figure 1:**
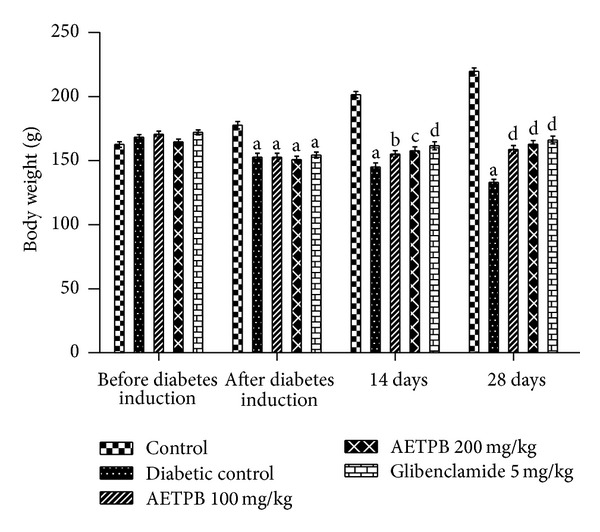
Effect of AETPB on body weight in STZ-NIC-induced diabetic rats. All data are expressed as mean ± SEM (*n* = 6). ^a^ *P* < 0.001 diabetic control, AETPB 100 and 200 mg/kg, glibenclamide 5 mg/kg compared with control; ^b^ *P* < 0.05 AETPB 100 mg/kg compared with diabetic control; ^c^ *P* < 0.01 AETPB 200 mg/kg compared with diabetic control; ^d^ *P* < 0.001 AETPB 100 mg/kg, AETPB 200 mg/kg, and glibenclamide 5 mg/kg compared with diabetic control.

Administration of STZ-NIC significantly (*P* < 0.001) increased the blood glucose level compared to normal control rats. A significant (*P* < 0.001) reduction of blood glucose level was noticed in type 2 diabetic rats after the oral administration of both doses of AETPB and glibenclamide than diabetic control rats (Table 1). The body weight of rats was reduced after STZ-NIC administration significantly (*P* < 0.001) than normal control rats (Figure 1). A significant (*P* < 0.001) increased body weight in diabetic rats was observed after AETPB 100 and 200 mg/kg and in glibenclamide administration when compared to diabetic control rats. 

#### 3.1.2. Serum Insulin, Hb, HbA1c, and TP Levels Changes in Type 2 Diabetic Rats

STZ-NIC-mediated diabetes induction in rats increases HbA1c levels and reduces serum insulin, Hb, and TP significantly (*P* < 0.001) when compared to normal control rats (Table 2). Oral administration of both doses of AETPB and standard drug glibenclamide to the type 2 diabetic rats showed significant (*P* < 0.001) reduction of HbA1c levels and increase in Hb, TP, and serum insulin levels than diabetic control rats. AETPB at 200 mg/kg dose showed significant (*P* < 0.001 and *P* < 0.01) higher efficacy than AETPB 100 mg/kg dose on normalization of Hb and HbA1c levels in type 2 diabetic rats.

#### 3.1.3. Lipid Profiles Changes in Type 2 Diabetic Rats

A significantly (*P* < 0.001) increased level of TC and TG and reduction of HDL level were observed after STZ-NIC-induced diabetic rats than normal control rats (Table 3). AETPB treatment significantly (*P* < 0.001) decreased TC, TG levels in diabetic rats and increased HDL level significantly (*P* < 0.05) when compared to diabetic rats treated with vehicle. Administration of AETPB at 200 mg/kg dose showed higher reduction in TC, TG levels and increase in HDL level than AETPB 100 mg/kg dose but failed to show statistical significance.

### 3.2. Characterization of Biomarkers

**Figure 2:**
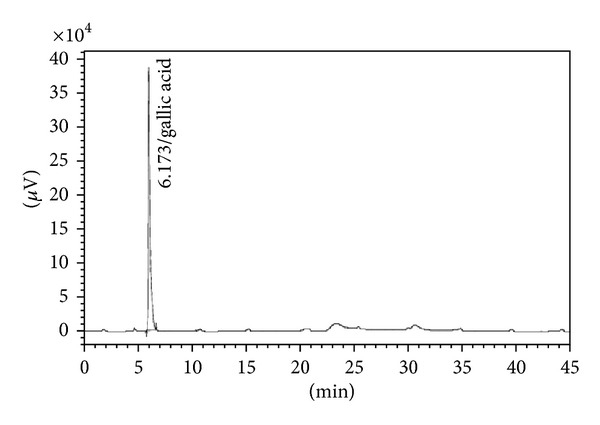
Chromatogram of standard gallic acid.

**Figure 3:**
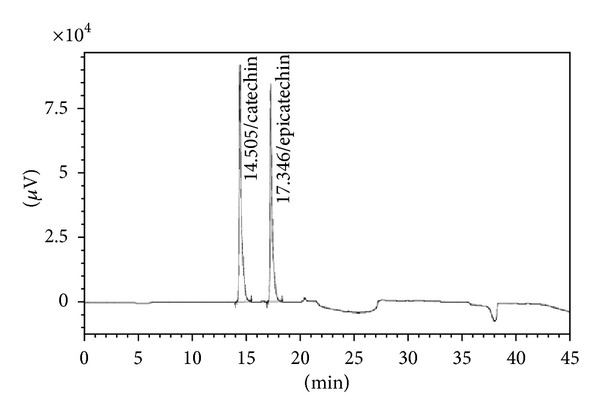
Chromatogram of standard catechin and epicatechin.

**Figure 4:**
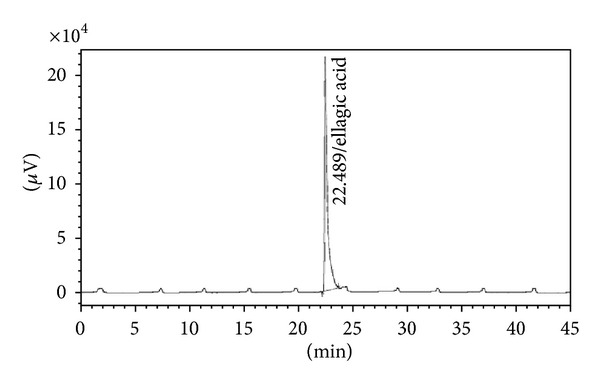
Chromatogram of standard ellagic acid.

**Figure 5:**
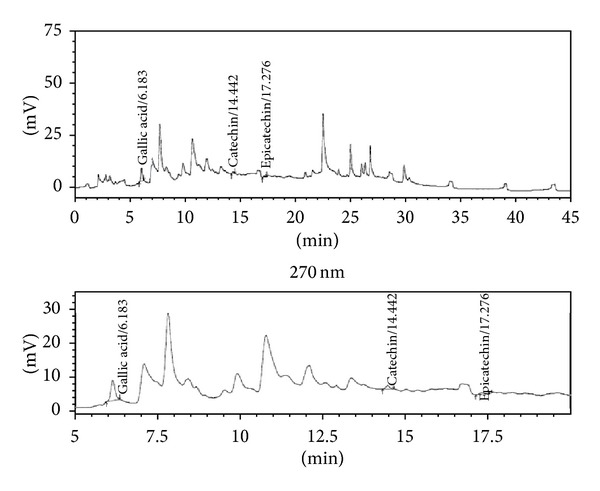
Chromatogram of AETPB for gallic acid, catechin, and epicatechin.

**Figure 6:**
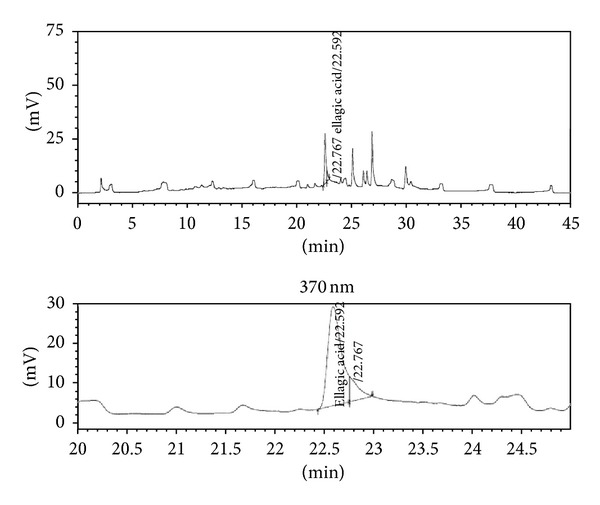
Chromatogram of AETPB for ellagic acid.

The preliminary phytochemical analysis confirmed the presence of secondary metabolites flavonoids and tannins in AETPB. In HPLC analysis, presence of biomarkers gallic acid, ellagic acid, catechin and epicatechin in AETPB was confirmed by comparison of their retention time (Figures 2, 3, 4, 5, and 6 and Table 4) with respective standard phytoconstituents retention time.

### 3.3. *In Vitro* Studies

#### 3.3.1. Cytotoxicity; Glucose Uptake Action of AETPB and GA in L6 Rat Skeletal Muscle Cells

Cytotoxicity assay on L6 rat skeletal muscle cells showed that AETPB and GA were non-toxic up to 200 *μ*g/mL and 100 *μ*M concentrations, respectively. In all tested concentrations a more than 80% cells viability were noticed.

In present study, insulin sensitization effect of AETPB and GA was examined in L6 rat skeletal muscle cells *via *glucose uptake action to confirm possible antidiabetic mechanism (Table 5). In this assay, incubation of AETPB (0.5, 5, and 10 *μ*g/mL), GA (0.05, 0.5, and 5 *μ*M), and metformin (0.01 mM) in muscle cells in the presence of insulin (1 *μ*mol/L) showed significant (*P* < 0.001) glucose uptake action when compared to vehicle control. The standard drug metformin enhanced glucose uptake activity higher than AETPB and GA in the presence and absence of insulin. AETPB and GA did not exhibit higher glucose uptake action like standard drug metformin in the absence and presence of insulin. But results confirmed that AETPB and GA enhanced glucose uptake activity in the presence of insulin than absence of insulin when compared to vehicle control. AETPB produced dose-dependent glucose uptake action. 

#### 3.3.2. Inhibition of *α*-Amylase Activity

AETPB produced 10.14% inhibition of *α*-amylase activity at 1 *μ*g/mL and 82.31% at 10 *μ*g/mL concentrations, respectively, and its IC_50_ was found to be 3.62 *μ*g/mL. The standard drug acarbose exhibited 14.67% inhibition of *α*-amylase activity at 50 *μ*g/mL and 93.49% at 1000 *μ*g/mL concentrations, respectively, and its IC_50_ for acarbose was found to be 219.50 *μ*g/mL (Table 6). 

#### 3.3.3. Inhibition of *α*-Glucosidase Activity

AETPB exhibited 5.91% inhibition of *α*-glucosidase activity at 25 *μ*g/mL and 85.36% at 1600 *μ*g/mL concentration, respectively, and its IC_50_ was found to be 287.10 *μ*g/mL. The standard drug acarbose produced 20.55% inhibitory effect on *α*-glucosidase activity at 0.1 *μ*g/mL and 91.37% at 3.2 *μ*g/mL concentrations, respectively, and its IC_50_ was found to be 0.39 *μ*g/mL (Table 7). 

## 4. Discussion

The screening of antidiabetic activity of natural products and synthetic compounds is performed in experimental animal models after induction of diabetes by several methods. To induce non-insulin-dependent diabetes in animals, streptozotocin-nicotinamide is commonly used which produces moderate hyperglycemia with clinical symptoms similar to type 2 diabetes [15]. STZ causes alkylation of pancreatic deoxyribonucleic acid by entering to the *β*-cell *via* glucose transporter 2 and induces activation of poly (ADP-ribosylation) that causes depletion of cellular nicotinamide adenine dinucleotide (NAD^+^) and adenosine triphosphate. As a result, generation of free radicals causes pancreatic *β*-cells necrosis [16]. Nicotinamide administration along with STZ can act as a weak poly (ADP-ribose) polymerase (PARP) inhibitor, which prevents activation of poly ADP-ribosylation, and precursor for the coenzyme NAD^+^ which is necessary for the cellular function and metabolism. Thus, nicotinamide prevents pancreatic damage caused by STZ-mediated cytotoxicity and produces a diabetic condition in rats similar to human type 2 diabetes [17]. In our study, induction of type 2 diabetes showed significant increased blood glucose level and decreased body weight and insulin level compared to control rats which confirm the induction of diabetes, and it may be due to partial necrosis of pancreatic *β*-cell by STZ. Also, body weight of diabetic rats was decreased, and it may be due to reduction of insulin. Physiologically, insulin regulates protein synthesis and proteolysis in skeletal muscle [18]. Oral administration of AETPB (100 and 200 mg/kg dose) and glibenclamide to the diabetic rats showed significant reduction of blood glucose and increase in body weight and insulin level than diabetic control rats. Hence, AETPB mediated above effect possibly due to its preventive effect on STZ-mediated *β*-cell damage in diabetic rats and thereby increases insulin release and inhibits muscle proteolysis which causes improvement in body weight of AETPB-treated diabetic rats. 

The alteration of lipid metabolism due to insulin resistance causes lipoproteins abnormalities which are commonly observed in type-2 diabetic patients, and it increases cardiovascular diseases associated with atherogenic dyslipidaemia. The persistent hyperglycaemia causes glycosylation of all proteins, specifically collagen cross linking and matrix of arterial wall which causes endothelial cell dysfunction and it further progresses atherosclerosis. The prevalence of dyslipidemia in diabetes mellitus is 95%, and it is a major risk factor for the development of coronary heart disease [19]. Insulin resistance has a central role in the pathogenesis of diabetic dyslipidemia which causes increased free fatty-acid release from insulin-resistant fat cells and promotes more triglycerides production [20]. Presence of hypertriglyceridemia and hypercholesterolemia and reduction of HDL are the most common lipid abnormalities that were reported in diabetic condition [19]. Our study results showed significant increases in serum TC and TG levels and reduction in serum HDL level in type 2 diabetic rats. Administration of both the doses of AETPB and glibenclamide decreased levels of TC and TG as well as increased HDL levels in diabetic rats than diabetic control rats. This action of AETPB will help to reduce or prevent macrovascular complications in diabetes.

The glycosylated haemoglobin is an important clinical marker in diabetes which helps to determine the degree of protein glycation during diabetes [21]. In persistent hyperglycemic state, formation of HbA1c occurred by nonenzymatic reaction between glucose and free amino groups of haemoglobin. HbA1c level in diabetes helps to evaluate long-term glycemic control, and it helps to assess the risk of the development or progression of diabetic complications. Published studies supported that reduction in HbA1c levels during the diabetes treatment considerably reduced microvascular complications [22]. In STZ-NIC-induced diabetic rats, significantly decreased Hb and increased HbA1c levels were noticed than control rats. AETPB treatment showed reduction of HbA1c and improvement in Hb levels, and it might be due to blood glucose lowering effect of AETPB possibly through reversal of insulin resistance or increasing insulin secretion by regeneration of pancreatic *β*-cells.

The key enzymes for carbohydrate metabolism in the small intestine are pancreatic *α*-amylase and *α*-glucosidase which convert consumed polysaccharides to monosaccharides. This enzyme action causes postprandial blood glucose level elevation due to absorption of formed glucose from polysaccharides in the small intestine. Drugs having an inhibitory action on both of these enzymes possess an ability to control of postprandial blood glucose level specifically in type 2 diabetic patients. Currently, available drugs in this category are acarbose and miglitol, which competitively inhibit above enzymes. But these drugs have common side effects such as flatulence and abdominal bloating [14, 23]. New drugs or formulations which are devoid of the above side effects will improve the compliance in type 2 diabetic patients. Our present study results clearly demonstrated that AETPB possesses potent pancreatic *α*-amylase and *α*-glucosidase inhibition which confirmed that *in vivo* antidiabetic action of AETPB may be due to inhibition of the above enzymes. 

In type 2 diabetes, peripheral insulin resistance and impaired insulin secretion from pancreatic *β*-cells are two important features. Insulin resistance in peripheral tissues such as liver, skeletal muscle, and adipose tissue is commonly observed. The occurrence of cardiovascular diseases in type 2 diabetic patients mainly due to insulin resistance mediated hyperglycemia and dyslipidemia. Drug which diminishes insulin resistance will effectively control hyperglycemia, normalize lipid metabolism in type 2 diabetes, and hence it will prevent the diabetes-mediated cardiovascular complications [24]. The drugs like metformin and pioglitazone will ameliorate insulin resistance and control the hyperglycemia and abnormal lipid metabolism. This class of drugs has adverse effects such as lactic acidosis, gastrointestinal disturbance, liver toxicity, and cardiovascular risk [25]. Thus, drugs which improve insulin sensitivity without adverse effects were reported to be useful for the long-term treatment in type 2 diabetes. In present study, we examined insulin sensitization action of AETPB and GA in L6 rat skeletal muscle cells, and its efficacy was compared with metformin. The results revealed that AETPB and GA showed insulin sensitization action *via* enhancement of glucose uptake in muscle cells. Metformin produced higher glucose uptake in skeletal muscle cells in the absence and presence of insulin when compared to vehicle control, AETPB, and GA. But glucose uptake action of AETPB and GA in muscle cells in the absence and presence of insulin was not as potent as metformin. This data has given clear evidence that AETPB possibly acts to improve the glucose uptake in skeletal muscle in the presence of insulin, and hence it has potential to reverse insulin resistance in type 2 diabetes.

Presence of phenolic compounds and tannins in AETPB was noticed in preliminary phytochemical analysis. Further, HPLC analysis confirmed that AETPB contains biomarkers such as gallic acid, ellagic acid, catechin, and epicatechin. Antidiabetic, antilipid peroxidative, and antioxidant activity of isolated and synthetic GA was documented in diabetic rats [26]. The GA, isolated from *Terminalia bellerica* fruit, treatment in diabetic rats showed significant antidiabetic activity [27]. GA-mediated glucose transporter 4 (GLUT4) translocation, glucose uptake in adipocyte cells, and insulin secretagogue actions in pancreatic cells were reported [28, 29]. In present study, insulin sensitization assay, incubation of AETPB and GA enhances glucose uptake in skeletal muscle cells which confirmed that GA possibly mediate the antidiabetic activity of AETPB.

**Figure 7:**
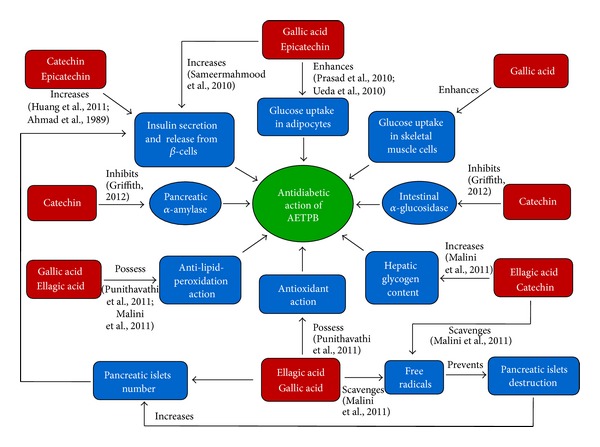
Proposed multiple mechanisms for AETPB antidiabetic activity.

The phytoconstituents such as catechin, epicatechin, and ellagic acid were reported for their *in vivo* and *in vitro* antidiabetic activity. Catechin showed significant antidiabetic activity in STZ-induced diabetic rats, and docking studies confirmed it might be due to activation of insulin receptor and agonist action on peroxisome proliferator-activated receptor gamma [30]. Also, catechin-mediated inhibitory effect on *α*-amylase and *α*-glucosidase was reported [31]. Catechin showed enhanced insulin secretion in presence of glucose and significant reversal of glucose intolerance in high-fat-diet-induced diabetic mice [32]. Epicatechin-mediated enhancement of glucose uptake activity in the absence of insulin *via* GLUT4 translocation in adipocytes in a dose-dependent manner was documented [33]. Moreover, epicatechin was reported to produce insulinogenic and insulin-like actions in a dose-dependent manner [34]. Also, ellagic acid was reported for the antioxidation, anti-lipid-peroxidation, and enhancement of hepatic glycogen content in diabetic rats [35]. Therefore, antidiabetic activity of AETPB might be due to presence of potential biomarkers such as gallic acid, catechin, epicatechin, and ellagic acid. Based on our findings and above reports, we have proposed that AETPB acts through multiple mechanisms (Figure 7) to control blood glucose in diabetes.

## 5. Conclusion

Aqueous extract of *Terminalia paniculata *bark has potential antidiabetic activity in diabetic rats. *In vitro* study results scientifically supported the *Terminalia paniculata *bark *in vivo* antidiabetic activity. Further, aqueous extract of *Terminalia paniculata *bark contains active biomarkers which may possibly be responsible for the antidiabetic activity of *Terminalia paniculata *bark.

## Figures and Tables

**Table 1:** Blood glucose lowering effect of AETPB in type 2 diabetic rats.

Groups	Dose (mg/kg)	Blood glucose (mg/dL)			
		Before diabetes induction (0 day)	After diabetes induction (14 days)	Day 14	Day 28
Control	Vehicle	59.50 ± 2.09	65.33 ± 1.76	68.33 ± 2.03	60.50 ± 1.87
Diabetic control	Vehicle	62.50 ± 1.89	251.50 ± 6.29^a^	272.00 ± 6.32^a^	303.50 ± 7.34^a^
AETPB	100	56.66 ± 1.92	260.83 ± 5.70^a^	157.00 ± 3.22^b^	92.67 ± 2.41^b^
AETPB	200	58.50 ± 2.32	254.33 ± 5.19^a^	155.17 ± 4.54^b^	85.33 ± 3.51^b,c^
Glibenclamide	5	60.00 ± 2.12	262.67 ± 8.71^a^	140.83 ± 3.23^b^	73.16 ± 2.57^b,c^

All data are expressed as mean ± SEM (*n* = 6). Vehicle: 0.2% CMC (5 mL/kg).

^a^ *P* < 0.001 diabetic control compared with control.

^b^ *P* < 0.001 AETPB 100, 200 mg/kg and glibenclamide 5 mg/kg compared with diabetic control.

^c^ *P* < 0.001 AETPB 200 mg/kg or glibenclamide 5 mg/kg compared with AETPB 100 mg/kg.

**Table 2:** Effect of AETPB on haemoglobin, glycosylated haemoglobin, serum insulin, and total protein in type 2 diabetic rats.

Groups	Dose (mg/kg)	Hb (g/dL)	HbA1c (%)	Serum insulin (*µ*IU/mL)	TP (g/dL)
Control	Vehicle	13.92 ± 0.15	5.90 ± 0.22	9.06 ± 0.17	7.21 ± 0.26
Diabetic control	Vehicle	7.31 ± 0.27^a^	12.03 ± 0.37^a^	4.95 ± 0.15^a^	4.95 ± 0.19^a^
AETPB	100	11.67 ± 0.25^b^	7.34 ± 0.20^b^	6.74 ± 0.21^b^	6.26 ± 0.22^b^
AETPB	200	10.83 ± 0.34^b^	8.52 ± 0.18^b^	7.11 ± 0.23^b^	5.80 ± 0.20^c^
Glibenclamide	5	13.17 ± 0.31^b^	6.89 ± 0.16^b^	8.17 ± 0.22^b^	6.85 ± 0.14^b^

All data are expressed as mean ± SEM (*n* = 6). Vehicle: 0.2% CMC (5 mL/kg).

^a^ *P* < 0.001 diabetic control compared with control.

^b^ *P *< 0.001 AETPB 100, 200 mg/kg and glibenclamide 5 mg/kg compared with diabetic control.

^c^ *P *< 0.05 AETPB 200 mg/kg compared with diabetic control.

**Table 3:** Effect of AETPB on lipid profiles in type 2 diabetic rats.

Groups	Dose (mg/kg)	Serum lipid profile levels (mg/dL)		
		TC	TG	HDL
Control	Vehicle	79.31 ± 2.51	87.63 ± 2.33	62.71 ± 1.36
Diabetic control	Vehicle	128.20 ± 3.01^a^	142.22 ± 4.33^a^	40.50 ± 1.67^a^
AETPB	100	75.46 ± 2.21^b^	83.08 ± 2.96^b^	56.76 ± 2.10^b^
AETPB	200	82.30 ± 3.05^b^	94.55 ± 3.39^b^	50.81 ± 1.63^c^
Glibenclamide	5	72.46 ± 2.66^b^	75.23 ± 3.06^b^	60.82 ± 2.19^b^

All data are expressed as mean ± SEM (*n* = 6). Vehicle: 0.2% CMC (5 mL/kg).

^a^ *P* < 0.001 diabetic control compared with control.

^b^ *P* < 0.001 AETPB 100 and 200 mg/kg and glibenclamide 5 mg/kg compared with diabetic control.

^c^ *P* < 0.01 AETPB 200 mg/kg compared with diabetic control.

**Table 4:** AETPB phytoconstituents and their retention time comparison with standard phytoconstituents.

Phytoconstituents	Amount (%w/w)	Retention time (Min)	
		Standard	AETPB
Gallic acid	0.044	6.173	6.183
Ellagic acid	0.134	22.489	22.592
Catechin	0.039	14.505	14.442
Epicatechin	0.023	17.346	17.276

**Table 5:** Glucose uptake in L6 rat muscle cells after 48 h incubation in media with glucose (2 g/L).

Treatment	Concentration	Glucose consumption (mg/100 mL)	
		Absence of insulin	Presence of insulin (1 *µ*mol/L)
Vehicle control	0.1% DMSO	2.95 ± 0.07	7.17 ± 0.03

Metformin	0.01 mM	7.80 ± 0.06^a^	8.96 ± 0.03^a^
	0.1 mM	4.84 ± 0.09^a^	7.68 ± 0.13^a^

AETPB	0.5 *µ*g/mL	4.77 ± 0.07^a^	7.69 ± 0.06^a^
	5 µg/mL	4.05 ± 0.08^a^	7.79 ± 0.05^a^
	10 *µ*g/mL	3.98 ± 0.07^a^	7.96 ± 0.04^a^

Gallic acid	0.05 µM	4.04 ± 0.05^a^	7.96 ± 0.09^a^
	0.5 *µ*M	3.96 ± 0.07^a^	7.87 ± 0.10^a^
	5 *µ*M	3.58 ± 0.14^a^	7.83 ± 0.09^a^

The data represented as mean ± SD (*n* = 3).

^a^ *P* < 0.001 metformin, AETPB, and gallic acid compared with vehicle control.

**Table 6:** Inhibition of *α*-amylase enzyme activity by AETPB.

Sample	Concentration (*µ*g/mL)	% inhibition of enzyme activity	IC_50_ (*µ*g/mL)
AETPB	1	10.14 ± 0.75	3.62
	2	40.99 ± 0.84	
	4	50.48 ± 1.30	
	6	60.95 ± 0.95	
	8	72.60 ± 1.31	
	10	82.31 ± 1.02	

Acarbose	50	14.67 ± 0.58	219.50
	100	24.30 ± 0.53	
	200	45.82 ± 1.03	
	400	70.05 ± 0.85	
	800	81.87 ± 0.72	
	1000	93.49 ± 0.69	

The data represented as mean ± SD (*n* = 3).

**Table 7:** Inhibition of *α*-glucosidase enzyme activity by AETPB.

Sample	Concentration (*µ*g/mL)	% inhibition of enzyme activity	IC_50_ (*µ*g/mL)
AETPB	25	5.91 ± 0.40	287.10
	50	14.03 ± 0.36	
	100	21.45 ± 0.42	
	200	47.32 ± 0.51	
	400	58.57 ± 0.58	
	800	70.41 ± 0.41	
	1600	85.36 ± 0.42	

Acarbose	0.1	20.55 ± 0.35	0.39
	0.2	41.77 ± 0.54	
	0.4	51.16 ± 1.06	
	0.8	60.83 ± 0.76	
	1.6	73.46 ± 1.04	
	3.2	91.37 ± 1.14	

The data represented as mean ± SD (*n* = 3).
